# Bowel problem management among nursing home residents: a mixed methods study

**DOI:** 10.1186/s12912-014-0035-9

**Published:** 2014-11-25

**Authors:** Susan Saga, Arnfinn Seim, Siv Mørkved, Christine Norton, Anne Guttormsen Vinsnes

**Affiliations:** Faculty of Nursing, Sør-Trøndelag University College, Postbox 2320, 7004 Trondheim, Norway; Department of Public Health and General Practice, Norwegian University of Science and Technology, Postbox 8905, 7491 Trondheim, Norway; Clinical Service, St. Olavs Hospital, Trondheim University Hospital, 7006 Trondheim, Norway; Florence Nightingale Faculty of Nursing and Midwifery, King’s College London, 57 Waterloo Road, London, SE1 8WA UK

**Keywords:** Constipation, Diarrhoea, Faecal incontinence, Nursing homes, Cross-sectional survey, Focus groups

## Abstract

**Background:**

Bowel problems such as constipation, diarrhoea and faecal incontinence (FI) are prevalent conditions among nursing home residents and little is known about nursing management. This study aimed to elucidate how Norwegian registered nurses (RNs) manage bowel problems among nursing home residents.

**Methods:**

A mixed methods approach was used combining quantitative data from a population-based cross-sectional survey and qualitative data from a focus group interview. In the cross sectional part of the study 27 of 28 nursing homes in one Norwegian municipality participated. Residents were included if they, at the time of data collection, had been a resident in a nursing home for more than three weeks or had prior stays of more than four weeks during the last six months. Residents were excluded from the study if they were younger than 65 years or had a stoma (N = 980 after exclusions). RNs filled in a questionnaire for residents regarding FI, constipation, diarrhoea, and treatments/interventions. In the focus group interview, 8 RNs participated. The focus group interview used an interview guide that included six open-ended questions.

**Results:**

Pad use (88.9%) and fixed toilet schedules (38.6%) were the most commonly used interventions for residents with FI. In addition, the qualitative data showed that controlled emptying of the bowels with laxatives and/or enemas was common. Common interventions for residents with constipation were laxatives (66.2%) and enemas (47%), dietary interventions (7.3%) and manual emptying of feces (6.3%). In addition, the qualitative data showed that the RNs also used fixed toilet schedules for residents with constipation. Interventions for residents with diarrhoea were Loperamide (18.3%) and dietary interventions (20.1%). RNs described bowel care management as challenging due to limited time and resources. Consequently, compromises were a part of their working strategies.

**Conclusions:**

Constipation was considered to be the main focus of bowel management. Emptying the residents’ bowels was the aim of nursing intervention. FI was mainly treated passively with pads and interventions for residents with diarrhoea were limited. The RNs prioritized routine tasks in the nursing homes due to limited resources, and thereby compromising with the resident’s need for individualized bowel care.

## Background

Faecal incontinence (FI) and constipation are prevalent conditions among nursing home residents [1,2]. The conditions cause difficulty with hygiene, skin problems, pain and discomfort, as well as having serious implications for residents’ dignity [3]. Bowel problems are a professional challenge for nursing home staff. In addition, FI is associated with high costs related to pad use, time spent by staff to wash and change patients, laundry expenses, skin damage and infections. A nursing home is a place of residence for people with health problems and significant deficiencies in activities of daily living (ADL), requiring medical or nursing care. Nursing home residents could be described as “frail older persons who have impaired physical activity, mobility, muscle strength, cognition, nutrition, and endurance” [2]. Nursing home residents are a particularly vulnerable population as they have lost the independence to live in the community through illness, disability and frailty. At the same time, recognition of the unique skill of nursing home staff is low [4]. Life expectancy will increase in the in the coming decades, however often with accompanying morbidities [5]. These demographic changes will constitute a significant challenge for the health care systems involved, particularly nursing homes. RNs spend a lot of time managing residents’ bowel problems in nursing homes and they have a key role in the assessment, prevention and management of bowel problems. However, there is little evidence on how RNs actually manage bowel problems and the quality of the interventions performed.

### Bowel problems in nursing home residents

FI is “the involuntary loss of liquid or solid stool that is a social or hygienic problem” [1]. Previous studies on FI in nursing home residents suggest a prevalence between 33.3% and 66.8% [6-9]. Constipation is defined as an abnormality of stool bulk, hardness or frequency and might be associated with straining [10]. It is however common to take laxative use as a marker for constipation in clinical studies. Prevalence of constipation is reported as between 50-74% in institutionalized elderly people in an evidence-based review [11]. Chronic diarrhoea is defined as the frequent passage of unformed stool for more than three weeks [12]. The prevalence of diarrhoea in nursing homes is rarely reported in scientific work and is mostly associated with studies on Clostridium difficile. Clostridium difficile-associated diarrhoea has been identified in 3.4% to 21.7% of nursing home residents [13-15]. In a population-based Norwegian cross-sectional study, RNs reported that 42,3% of residents had involuntary leakage of faeces once a week or more often, 41.0% of the nursing home residents were constipated when laxative use was included as a marker, and the prevalence of diarrhoea was 17.3% [9].

Bowel symptoms such as constipation and diarrhoea are related to FI, as well as laxative use [1]. Among frail elderly people, FI is also associated with reduced mobility, cognitive impairment, co-morbidity, urinary incontinence [16] and length of stay in the nursing home [9]. Constipation among elderly people is related to loss of mobility, medications, underlying diseases, impaired anorectal sensation, and ignoring calls to defecate [17]. Low fibre intake and limited fluid intake have also been implicated as causes of constipation, but there is little evidence from the literature to support this [18]. Diarrhoea is related to infections, faecal impaction/constipation, co-morbidity or side effect of medications [19].

Clinical guidelines for both community dwelling and frail elderly patients with FI, constipation or diarrhoea are available [1], but not for the group of institutionalized elderly. Bowel problems among nursing home residents are multifaceted conditions which are often inter-related, and treatment and care for these conditions will often require a multifactorial intervention approach. The International Consultation on Incontinence (ICI) recommends that all patients in nursing homes should be assessed for faecal incontinence [1]. Treatment and care for residents with bowel problems should be directed to underlying causes and be evidence-based. The ICI’s recommendations for frail elderly with constipation which has not responded to lifestyle modifications such as toileting as needed, diet and mobility are to achieve an effective bowel clearance by using a combination of laxatives and enemas, followed by maintenance therapy with laxatives or suppositories [1,16]. A previous study has demonstrated that chronically constipated patients improved or became symptom free by using supplemental fiber [20]. There is however no evidence that increased fluid intake improves constipation symptoms [17,18]. Loperamide has shown good effect in chronic diarrhoea, but should be used with caution due to possible mis-interpretation of spurious diarrhoea caused by faecal impaction [1]. Also, dietary measures such as soluble dietary fibre [1] and probiotic yogurt drinks [21] are recommended.

There are few published trials of treatment for FI in older people, and the few existing studies mostly have small numbers and problematic methodology [1]. Conservative treatment such as bowel training [22], biofeedback treatment [23] and pelvic floor training [24] has shown some effect among frail elderly patients with FI. Other conservative treatment methods used, but with a more uncertain effect, are rectal irrigation, electric stimulation and using pads or an anal plug. In addition, it is important to modify stool consistency [25]. Detailed medical history on medications and co-morbid problems, rectal examination, colonoscopy and physiological tests may identify organic and functional causes. However, in frail older people, there is usually more than one mechanism, requiring an individualized but multifactorial treatment approach. The importance of identifying treatable causes is strongly emphasized, in addition to assessing the patients’ cognitive and functional level [1].

A recent systematic review on the management of practice and staff experience of urinary and faecal incontinence in nursing homes demonstrated that there were few studies available on FI practice, although some studies included both urinary incontinence and FI [26]. We have found no evidence on how RNs solve problems when caring for other bowel problems in nursing home residents. Bowel problems among nursing home residents are, to a large extent, related to remediable factors, and may for many residents be prevented and/or improved. The high prevalence of bowel problems among nursing home residents suggests a considerable potential for improvement within this group. Current nursing home practices do not adequately address these challenges. Hence, the present study aims to explore how RNs manage bowel problems among nursing home residents, in order to understand their clinical reasoning and decision making processes. This work is part of a larger research programme that aims to examine prevalence, correlates and management of FI among nursing home residents in Norway [9,27].

## Methods

In order to obtain information about how RNs manage bowel problems among nursing home residents, cross-sectional data were collected. RNs filled in a questionnaire regarding bowel interventions for all nursing home residents in one Norwegian municipality. In addition, in order to shed some light on the quantitative data, a qualitative focus group interview with RNs was used. A focus group interview is a technique used to obtain dynamic and interactive discussion to clarify the informants’ perceptions and experiences and is especially useful to reveal shared understanding and practice [28]. Using a mixed method approach, descriptive data from the population based cross-sectional study, was combined with rich qualitative data from a focus group interview to offer a more comprehensive picture of RNs’ bowel management practices in nursing homes [29]. This study was carried out in accordance with Declaration of Helsinki and The Regional Committee for Medical and Health research Ethics, REC South East, approved the study (2009/1225). No consent from residents or their next of kin was required by the ethical committee because all the resident information gathered was de-identified and anonymous to the researcher. The participants in the focus group interview gave their written informed consent to participate in the study.

### Setting

The cross-sectional study was performed in nursing homes in Trondheim municipality, Norway during June 2010 and the focus group interview was conducted in April 2013. Population-wise, Trondheim is the third largest municipality in Norway, and comprises both urban and rural areas. In Norway, the municipalities have a statutory obligation to provide nursing home services to those who need it [30]. As of 2010, there were 28 nursing homes in Trondheim. Most of them were run and owned by the municipality. However, there were also a few non-profit private providers. The nursing homes have RNs on duty 24-hours a day, and approximately 25% of the staff on each shift were registered nurses, 50% were licensed practical nurses and 25% were unskilled labour. Additionally, a physician is employed and has medical responsibility for the nursing home residents, but is only available for a few hours a week [31]. The staff-to-resident ratio was approximately 1:4 in most nursing homes on the day shift, 1:8 on the evening shift and fewer on the night shift. The total number of nursing home residents in the municipality was 1322 at the time of the study.

### Sample/participants

#### Sample 1 – nursing home residents

In the cross-sectional study all 28 nursing homes in one Norwegian municipality were invited to participate. Residents who were resident in a nursing home at the time of data collection were included if they had been a resident for more than three weeks or had prior stays of more than four weeks during the last six months. Residents younger than 65 years or who had a stoma were excluded from the study after data collection.

#### Sample 2 – RNs

The participants for the focus group interview were recruited through convenience sampling, and included 7 women and one man from 7 different municipal nursing homes. Mean age of the informants in the focus group was 40.9 years (range 26–61 years), mean years of work experience were 10.4 (range 3–40 years). Four of the RNs had postgraduate education in elderly care; only two of the RNs stated that they had received some degree of training regarding FI.

### Data collection

The cross-sectional study aimed to survey the nursing management of FI, constipation and diarrhoea. There were no existing survey instruments or questionnaires translated and validated in Norwegian for this purpose. Consequently, a questionnaire specifically designed for this study was developed to obtain information about: the residents’ admission date, type of care, birth year, sex, stoma, medical diagnosis, medications, cognitive impairment, FI, urinary incontinence, constipation, diarrhoea, and bowel management (FI, constipation and diarrhoea). An interdisciplinary expert group developed the questionnaire. The questionnaire was pilot tested in one nursing home unit [9,27]. To obtain information about the residents’ functional level, the validated Barthel’s ADL index was included [32]. RNs with comprehensive oversight of each patient filled in a questionnaire for each nursing home resident. The municipality received payment as a compensation for the time used for data collection. The methods for the questionnaire study are more thoroughly described elsewhere [9]. Questions regarding bowel interventions were based on recommendations from the consensus literature [1], previous studies [16-18,20-24] and common practice in Norwegian nursing homes and response options are presented in Table 1.

**Table 1:** **Frequency of interventions for residents (N = 980) with FI**, **constipation and diarrhoea**

**Intervention**	**FI (N = 415)**	**Constipation (N = 396)**	**Diarrhoea (N = 164)**
**No interventions (despite a reported problem)**	46 (11.1%)	81 (20.5%)	104 (63.4%)
**Pads pr 24 hours (total)**		-	-
0	46 (11.1%)	-	-
1-2	46 (11.1% )	-	-
3-4	277 (66.7%)	-	-
>5	46 (11.1%)	-	-
**Fixed toilet schedules**	160 (38.6%)	-	-
**Dietary interventions**	-	29 (7.3%)	33 (20.1%)
**Oil enema**	-	29 (7.3%)	-
**Tap water saline enema (isotonic)**	-	1 (0.3%)	-
**Small enema (Klyx®)** ^**1**^	-	135 (34.1%)	-
**Manual emptying of faeces**	-	25 (6.3%)	-
**Laxatives (tablets, oral liquid and suppositories)**	-	262 (66.2%)	-
**Anti-diarrhoeal medication**	-	-	30 (18.3%)
**Rectal irrigation**	8 (1.9%)	-	-
**Anal plug**	0	-	-
**Pelvic floor exercises**	0	-	-
**Biofeedback**	0	-	-
**Electro**-**stimulation**	0	-	-
**Free text remarks**	4 remarks:	22 remarks:	5 remarks:
	• Remind to go/offer resident assistance to toilet: (1)	• Hospital admission: (1)	• Cessation/reduce laxatives: (3)
	• Regime where bowel is emptied medically: (3)	• Micro enemas: (21)	• Bowel emptying due to spurious diarrhoea: (2)

^1^Contains docusate sodium and sorbitol.

The researchers contacted the head nurses in nine nursing homes in the municipality about participation in the focus group study. The head nurses selected RNs for the study according to convenience. The group met once at the local University College and the interview lasted for one and a half hours using an interview guide that included six open-ended questions:

1. Can you tell us about yourself and your role in the nursing home?
2. What do you do during your work day when you find that a resident has problems with hard and irregular bowel movements?
3. What do you do during your work day when you find that a resident has problems with diarrhoea or loose stools?
4. What do you do during your work day when you find that a resident has problems controlling bowel movements?
5. How can we make things better? (Specific expectations)
6. Is there anything that we have not discussed that you think is important to mention?

These topics were guided by the previous questionnaire responses. Two nurse researchers, both female and from the local Faculty of Nursing, facilitated the focus group interview: A PhD student (SS) moderated the group, which included keeping the discussion on track, ensuring that everyone took part, and balancing the participants’ contributions. The other researcher, a Professor (AGV), was present as an observer with responsibility to ensure that all six questions were discussed [28]. Both researchers had prior experience of conducting research interviews or focus group interviews.

### Data analysis

Statistical methods included estimating prevalence in percentages, chi-square test and other descriptive statistics. Statistical calculations were performed using PASW® statistics 19 for Windows (SPSS Inc., Chicago, Illinois USA).

The methodological orientation used to underpin the qualitative part of the study was a descriptive content analysis [33]. The focus group interview was audio-recorded and transcribed verbatim, retaining frequent repetitions, pauses, and emotional expressions [33]. In order to acquire an overview of themes and a general impression, the transcripts were first read through several times by two researchers independently (SS & AGV). The analysis then moved into meaning condensation and coding. Themes were partly identified in advance from the topic guide and partly derived from the data. The subsequent codes and sub-codes were categorized into an index manually. Finally, by comparing and contrasting the content in each category, meaning categorization was achieved [33]. In order to ensure the trustworthiness of the analysis, the coding process was cross-validated by the same two researchers who took part in the interview. In this mixed-methods study, an explanatory design was used where the development of a qualitative design was based on the quantitative results [29]. The data analyses were performed separately, and the findings were not compared until the interpretation stage. Although inferences were drawn after each phase (quantitative and qualitative), a meta-inference was drawn at the end of the interpretation phase, when the inferences from both designs were compared.

## Results

### Cross-sectional data

**Figure 1:**
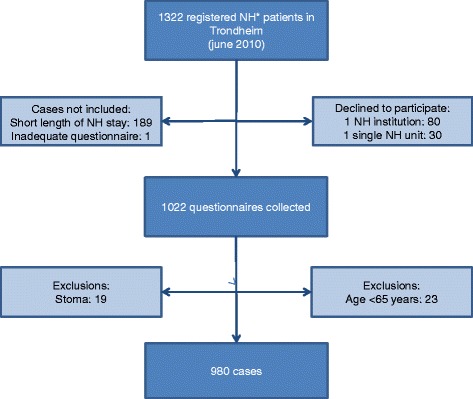
**Flow chart of inclusions and exclusions.** *Nursing home. Saga et al. [9].

Of the 28 nursing homes in a Norwegian municipality, 27 nursing homes participated. After exclusions 980 residents were included, a 90.3% overall response rate for the total municipal nursing home population (Figure 1). Mean age of the residents was 85.5 years (SD 7.3, range 65–107 years); 73.9% women and 26.1% men; 92.5% in long-term care and 7.5% in short-term care. The latter included rehabilitation and respite stays. Mean duration of stay was 881.9 (SD 871.0) days in long-term care, and 51.1 (SD 56.6) days in short-term care. Cognitive impairment was reported in 80.3% of the residents, and mean score on Barthel’s ADL index was 9.5 (SD 5.6). The RNs reported that 42.3% of residents had involuntary leakage of feces a few times a month or more often; 41% had constipation-related problems (including laxative use) according to the RNs’ assessment; 17.3% had diarrhoea or loose stool. Detailed results have been reported previously [9]. A total of 74.4% of the residents with FI used pads for FI, urinary incontinence or both; 59.3% of all the nursing home residents received oral laxatives regularly and 1.2% received micro enemas regularly.

Among the nursing home residents with constipation 66.2% received laxatives (tablets, oral liquids and suppositories), 34.1% were given enemas with 120 ml or 240 ml docusate sodium and sorbitol, 0.3% were given isotonic saline enemas (with tap-water), and 7.3% were given oil enemas for constipation. Nutritional interventions were used in 7.3% of the residents, while manual emptying of the bowels was used for 6.3%. No interventions were reported for 20.5% of the residents with constipation (Table 1). Additionally, there were 21 open responses regarding use of micro enemas (5.3%) for residents with constipation.

Loperamide was given to 18.3% and dietary interventions were carried out for 20.1% of the residents with diarrhoea. No interventions were carried out for 63.4% of the residents with diarrhoea or loose stool. Additionally, there were 5 open responses regarding diarrhoea, 3 of which concerned cessation or reduction of laxatives and 2 remarks on the need to empty the residents’ bowels due to spurious diarrhoea.

Most of the interventions for FI were related to pad use; 88.9% of residents with FI used one pad or more per 24 hours. Pads were used for both urinary and FI. Other interventions for FI were fixed toilet schedules, which were used in 38.6% of residents with FI and rectal irrigation used in 1.9%. No interventions were carried out for 11.1% of the residents with FI. Anal plugs, pelvic floor exercise, bio-feedback and electro stimulation were also response options in the questionnaire, but did not generate any responses. In addition, 4 open remarks were made regarding FI. Three of these remarks mentioned that the residents’ bowel was emptied medically in order to maintain control and avoid accidents. The remaining remarks reported the need to remind residents to go to the toilet, or to offer toileting assistance.

### Focus group data

From the survey responses six questions were developed (Box 1). The RNs were asked to talk about their experiences with bowel problems and FI among nursing home residents. Two overarching domains emerged from the focus group; firstly, the RNs described the challenges of providing good bowel care for this resident group (Table 2). Secondly, they described different solutions for managing bowel problems among nursing home residents (Table 3). Verbatim quotes are presented in italics.

**Table 2:** **Typology of RNs**’ **experience of bowel care management**: **challenges in bowel management**

**Category**	**Problem**	**Consequence(s)**
**Challenging resident group**	Immobility	-Inactivity
		-Cannot reach toilet in time
		-Suppression of the need to defecate
	Bedridden	-Unable to sit during defecation
	Cognitive impairment	-Cannot find toilet
		-Cannot communicate needs or problems
		-Take off pads although they are needed
	Heterogeneous resident group	-Require different approaches
	Monitoring problems	-Residents go alone to the toilet
	Dehydration	-Constipation
	Medication	-Pain- killers may cause constipation
		-Extensive use of laxatives may lead to intestines unable to function without laxatives
**Resident and family experience**	Faecal leakage or accidents	-Does not bother residents with dementia
		-Anxiety attack
		-Shamefulness
		-Attention-seeking behavior
		-Pads as safety
	Constipation	-Residents are restless or jumpy
	Family	-Some family will not talk about bowel problems
		-Residents do not want family to know about it
		-Next of kin have occasionally found their loved-ones in a mess with faeces
**Physical and organizational working conditions**	Staff shortage	-Busy working days with many tasks
		-Toileting is time-consuming
		-Cannot follow resident to the toilet in time
		-Good bowel routines have a low priority
	Staff discontinuity	-Discontinuity in resident contact
		-Unskilled nursing aides
		-Poor recording of bowel movements, fluid intake etc.
	Impractical physical environment	-Makes toileting difficult
		-Makes recording of bowel movements difficult
**Professional challenges for the nursing group**	Pre-conceptions	-Advanced age equated to FI
		-Constipation is considered to be a significant problem
		-Diarrhoea and FI are not considered to be significant problems
	Pad use	-Residents have been waiting for help from staff so many times
		-Residents do not want to bother busy nurses
		-Residents have got used to defecating in pads
	Interdisciplinary cooperation	-Nurses often alone in decision-making and initiative to treat
	Unfamiliar with FI treatment	-Never heard about electrical stimulation or biofeedback
		-Just barely heard about residents receiving surgery
	Care organization	-Primary nursing is viewed as positive for bowel management

**Table 3:** **Typology of RNs experience of bowel care management**: **solutions**

**Category**	**Constipation**	**Diarrhoea**	**FI**
**Problem solving tasks/prevention**	-Assess causes of constipation	-Assess causes of diarrhoea	-Fixed toilet times to prevent accidents
	-Laxative use for prevention and emptying bowels when needed	-Give products with probiotic yogurt drinks	-Controlled emptying of bowels with laxatives to avoid accidents
	-Recording of bowel movements and fluid intake	-Avoid milk products	-Pads as safety
	-Available drinks	-Offer the residents probiotic yogurt drinks	
	-Linseed and probiotic yogurt drinks	-Cessation of laxatives	
	-Fibre (fruits, berries, prunes)	-Administering Loperamide	
	-Mobilization	-Offer regular and nutritious meals	
	-Enemas for emptying bowels		
	-Fixed toilet schedules		
	-Give residents plenty of time in the toilet		
	-Give residents privacy in the toilet		
**Compromises**	-Use of bedside lift to get bedridden residents up in a sitting position on the bed or to the toilet although it is uncomfortable and humiliating		-Use of pads as safety although not always necessary
	-Recording of bowel movements is important, not where or how it is recorded.		

### Challenges in bowel management

In the “challenges in bowel management” domain, four categories were generated: 1) challenging resident group, 2) resident and family experience, 3) physical and organizational working conditions and 4) professional challenge (Table 2).

### Challenging resident group

“*It is very difficult in our nursing home because there is a lot dementia*. (…) *More than 80 percent have dementia diagnoses at different levels*, *so it*’*s not so easy for them to explain themselves*” (participant 2). The RNs described a situation where many residents were cognitively and/or physically impaired or had psychiatric diagnoses, thus requiring different approaches to bowel care: residents with dementia were unable to find the toilet or unable to communicate needs and residents with physical impairment had difficulties reaching the toilet in time. The RNs also experienced difficulty in obtaining an overview of bowel habits for residents who went to the toilet independently. The RNs experienced constipation as a considerable problem among nursing home residents which they felt was due to long-term use of laxatives, dehydration, pain-killers, inactivity, suppression of the need to defecate, or inability to sit during defecation.

### Resident and family experience

“*Then there*’*s the elderly residents with progressing dementia*, *they do not react at all*. (…) *They do not even know that they hold stool. If you do not notice the smell*, *they* [*the residents*] *do not respond to it*” (participant 8). Some RNs believed that residents with dementia did not care if they had faecal leakage, but other had experienced that residents felt shame or had anxiety attacks after faecal accidents. One RN felt that faecal accidents sometimes were a way of getting the staffs attention. The RNs described that some residents used pads for safety while others were ashamed to wear pads. One RN was concerned about residents being unable to communicate, feeling that restless or jumpy residents are often constipated. The RNs reported that many residents had reservations regarding staff talking about their bowel problems with their families. They also felt that the families were reluctant to discuss the topic, unless some of the next-of-kin were nurses themselves.

### Physical and organizational working conditions

“*I also think that there shouldn*’*t be too many people* [*working*] *with the resident within a given time. Then you*’*ll have a better overview* (…); [*It is better if*] *there are full time nurses or other professionals present on a daily basis*, *making observations together and managing to follow up on them*” (participant 3). The RNs stressed that there were too few staff, too many were unskilled and that they experienced a discontinuity in resident contact. The RNs felt that they spent a lot of time toileting residents, but still were not able to follow residents to the toilet in time and that recording bowel movements was not prioritized. The RNs also experienced that an unsuitable physical environment was a hindrance to good bowel care and accurate record-keeping: “*Our buildings are so awkward*, *if you have to run to the first floor*…*maybe you are far over there*, *then you have to go back here*, *then you have to go to the third floor*, *and then you have to go somewhere to record it*…*it may easily be forgotten*” (participant 7).

### Professional challenges for the nursing group

During the interview, the RNs considered constipation to be a significant problem, whereas diarrhoea and FI were not. They expressed great concern regarding the assessment of constipation, as well as recording the resident’s bowel movements. However, residents with diarrhoea and FI were not always examined. The RNs felt that FI was normal in advanced age among the residents. One RN expressed disagreement; she had a female resident of over a hundred years who was continent. The RNs felt that residents got used to defecating in pads, due to both having to wait for help, as well as not wanting to bother the nursing home staff. Furthermore, the RNs described a process during which defecating in pads gradually became a habit, signifying a shift in the personal boundaries of the residents when admitted to a nursing home: “*Unfortunately*, *I usually say that if people do not use pads when they come to us*, *just give us a month or two. Then they have become pad users for both one and the other* [*urine and feces*]. (…)… *It becomes a habit to be sitting with a pad*” (participant 2). The RNs also described how the GPs often entrusted the RNs with the initiative to start treatment for constipated residents. “*Our physician tells us to decide. We are more experienced than she is. So*, *we just inform her* [*the physician*] *about what we think is best*” (participant 4). They described poor interdisciplinary collaboration regarding bowel problems and FI in general. The RNs had never learned about electro-stimulation or biofeedback and surgical interventions as possible treatment alternatives for FI. The RNs believed that organization of daily work routines according to primary nursing principles would have had a useful impact on bowel management.

### Solutions

In the “Solutions” domain, two categories were generated: 1) problem solving tasks/ prevention, and 2) compromises (Table 3).

### Prevention and problem solving tasks

“*If* [*the resident*] *has been constipated for a long time*… *perhaps give them a micro enema*… *or give them laxative tablets on a regular basis. We are using Toilax*® [*stimulant laxative*] *a lot*” (participant 8). The RNs were very concerned about prevention of *constipation* and their ability to record bowel movements and fluid intake during their daily routines. Laxative use, easy accessible drinking water, fibre and mobilization were also common among the RNs’ approach to prevent constipation. All the RNs described extensive use of laxatives and medicated enemas. Fixed toilet schedules during the day were mentioned by some informants. Two RNs stressed the residents’ need for sufficient time and privacy in the toilet in order to be able to empty their bowels. Dietary interventions and sufficient fluids were mentioned, as well as the importance of assessing the causes of constipation.

The group stressed the importance of assessing the causes of *diarrhoea*, but reported that this was not always done. Cessation of laxatives, administering Loperamide, giving probiotic yogurt drinks instead of milk and regular and nutritious meals were mentioned as possible measures to improve diarrhoea. For prevention of *FI* the RNs felt that fixed toilet schedules had a good effect. They could also prevent accidents by using laxatives to control when bowels were emptied: “*I think it is very important to have fixed toileting schedules* (…). *Then you can also regulate it* (…) *by laxatives. That you are sort of able to regulate* (…) *to a certain extent* (…) *when they*’*re going to be emptied* [*the bowels*] … *to prevent* (…) *it from going wrong*” (participant 3). The RNs said that pad use was the most common way to prevent accidents.

### Compromises

“*I have also been involved in* (…) *that we have suspended* [*the residents in a hoist*]. *It is not our first option*, *but* (…) *if one is not successful when trying* [*to empty the resident*’*s bowels*], *you do everything you can. But anyway*, *I think that getting up in a sitting position*… *none of us lie down when we are going to*…[*defecate*] *either*. (…) *At least being able to lean forward and press instead of lying on your back*” (participant 5). Some RNs explained how they gave bedridden or immobile residents a laxative and then, after a while, suspended the resident above the bed over a bedpan using the bed-side lift. The sling made it possible for the resident to come up into a sitting position over the bed without heavy lifting. Some of the informants expressed that this practice was undignified for the resident. The RNs also described residents using pads although they did not need them. “*If they*’*re wearing pads* (…) *then they can wait a while longer*” (participant 1).

## Discussion

The quantitative data demonstrated that the use of pads and fixed toilet schedules were the most commonly used interventions for FI (Table 1). Additionally, the qualitative data showed that controlled emptying of the bowels (using laxatives and/or enemas) was used (Table 3). These findings are in accordance with results from the few studies existing on this topic; namely that toileting and pads were commonly used by the nursing home staff [26]. In the questionnaire used in this study, the RNs answered questions regarding what kinds of remedies/interventions were used for incontinence, thereby including both urinary and FI. However, in the focus group interview we did not focus on urinary incontinence. Pads were used for both urinary and FI and it is not likely that the RNs would be able to differentiate pad usage between the two. In addition, it seems likely that the RNs did not only have defecation in mind when the residents were toileted at fixed schedules, but also bladder emptying. This reveals that nursing is complex to measure and that it is not always easy to isolate specific nursing actions.

Regarding constipation, the quantitative data demonstrated that laxative use and enemas were common measures, as well as dietary interventions and manual emptying of faeces. In addition, the qualitative data showed that the RNs also used fixed toilet schedules to prevent constipation. Regarding diarrhoea, the quantitative data demonstrated that the RNs mainly managed the residents’ diarrhoea with Loperamide and dietary interventions. The qualitative data demonstrated the RNs’ reflections and considerations that were a part of their daily life regarding residents’ bowel problems; the importance of assessing probable causes of constipation and diarrhoea and the recording of bowel movements in residents with constipation. In addition, the RNs described bowel care management as challenging due to a challenging resident group, the impact of residents’ and families’ experience, physical and organizational working conditions and a professional challenge to nurses (Table 2). Over 60% of the residents with diarrhoea or loose stool had no reported interventions directed towards this problem. FI was mainly treated passively with pad use. The qualitative data revealed that the RNs did not consider diarrhoea or FI to be significant problems, despite the high prevalence of both as shown in the quantitative data. However, constipation was considered to be a great problem and both the quantitative and qualitative data showed that most of the RNs’ effort was directed towards solving this problem. There seemed to be a shared understanding that emptying resident’s bowels to prevent constipation was the main concern of the RNs. How this was done was less important, consequently leading to compromises in nursing ethical standards such as maintaining residents’ dignity.

The staff identified a challenging resident group and did not feel that they had enough time, resources and the physical and organizational conditions necessary to meet all the residents’ different needs. The RNs were doing their best to manage the resident’s needs within limited time and resources. Nevertheless, all nursing activities are performed within a physical and organizational framework and to have a personalized care approach requires that this is founded in the institutional leadership [34]. The management of bowel problems and FI will therefore require an institutional commitment. The care quality is dependent on the competence of the staff and the standards they set. RNs are in charge of the nursing care given in nursing homes through guidance of other skilled and unskilled staff, including bowel care. In the setting for this study, only 25% of caring staff were RNs and this may pose a limitation for the quality of the care. In the meanwhile, pads are the most widely used intervention for FI; a very passive solution which may enhance the resident’s experience of being infantilized, and compromise their feeling of well-being. Not being able to control one’s bowel will likely undermine self-esteem and sense of dignity. The RNs in our study were also concerned about not being able to help residents to the toilet in time would make residents incontinent. Even if the residents felt the need and asked for help from the staff, they did not necessarily get help when they needed it. Robinson found in a qualitative study of nursing home residents that fear of being alienated by caregivers because of extra care required by toileting led to residents preferring or accepting the use of pads [35]. According to the RNs in our study, pads were considered a safety mechanism by both RNs and the residents who would ask for them. This dissonance causes stress because it leads to an ethical dilemma between what you think you should do and what you actually do.

This situation demonstrates that there is a great potential for improvement in continent emptying of bowels among nursing home residents. Given the fact that the RNs are responsible for the care given, and thereby quality of care in the nursing homes, RNs unique contribution is that they set the standard of care by their priorities, decisions and standards. It is their mandate to provide professional guidance to both skilled and unskilled staff. FI is a costly and debilitating condition, it is therefore important to prolong and maintain such an important function as continent emptying of bowels, which should be the RNs’ aim for good bowel care. In order to achieve this, an individual assessment is important. RNs should identify treatable causes of constipation, diarrhoea and FI, such as modifying stool consistency [16]. Although clear clinical guidelines are missing, it is of great importance that RNs implement existing evidence-based practice [1,16-18,20-25]. It is also important that RNs prioritize care needs differently, recognising the value of residents’ continence and providing toilet assistance in a timely manner. Education of health care workers should embed both a sense of value in identifying bowel problems, as well as a confidence that the conditions are treatable. This in turn depends upon institutional leadership that recognizes the importance of continence and bowel health. Toileting is time-consuming and requires resources. More importantly, continence and bowel care requires clinical competence in this specific field. Educational institutions for nurses should take responsibility for providing future nurses with sound knowledge of relevant care practices.

Although there is a need to establish new reliable knowledge regarding the management of FI among nursing home residents, there are few published trials of treatment of FI among nursing home residents, and no trials on prevention. The existing studies have small numbers, problematic methodology, and are all non-blinded [1]. This evidence on how RNs’ are actually managing FI in nursing homes will be an important contribution in the planning of future studies of interventions regarding FI or other bowel problems in nursing home.

### Strengths and limitations

In management of bowel problems it is important to focus on prevention as well as finding the best solutions for the problems that have evolved. However, this study focuses only on management when the resident already has bowel problems. Nevertheless, the RNs in the focus group interview were concerned about prevention of bowel problems, in particular prevention of constipation. The prevention perspective of bowel problems is therefore present in this study, although this was not the intended aim of the study. Due to multiple morbidity and high prevalence of cognitive impairment in the targeted population, proxy data from RNs was used in this study.

The strength of this study is the robust population-based cross-sectional methodology. The quantitative data showed that management was inadequate and restricted to a few methods of handling bowel problems in nursing home residents. In order to shed light on the quantitative findings we conducted a focus group interview with RNs. Management of bowel problems among nursing home residents is a matter of interaction between co-workers, and in a focus group interview the shared experience, perception and views would be highlighted. The qualitative focus group design was intended to gain a broader understanding of RNs’ care for nursing home residents’ FI, and we wanted a dynamic and interactive discussion in order to clarify their perceptions. Despite the limitation of having only one focus group interview, the strength of this study was its use of mixed methods. By using a combination of quantitative data enlightened by qualitative data, rich data were provided on how RNs manage bowel problems in nursing homes. The participants for the focus group interview were a convenience sample. It is however unlikely that the sampling is biased: the participating RNS were not chosen or selected based on characteristics, interests or views. They were the RNs on duty on the day of the interview. The participating RNs are therefore likely to be representative of the other RNs working in the nursing homes in the municipality.

Nursing homes are heterogeneous across the world and international comparisons are difficult to make [36]. Nevertheless, we believe that this nursing home study from one Norwegian municipality has an international interest and concern. Despite variations in nursing home organization and practice across the world, nursing home residents are frail elderly in need of professional care. The high prevalence of bowel problems is one of the characteristics of this group. RNs throughout the world are familiar with limited time and resources in their practice. Therefore, despite local/national practice variations, we believe that most of the practices described in this study will be applicable to nurses in other countries.

## Conclusions

In this mixed methods study of RNs’ management of bowel problems and FI in nursing home residents, we found that FI and diarrhoea were not considered to be significant problems, despite the high prevalence of both. Due to too many tasks and too few resources, the RNs were not able to complete tasks effectively. In this situation, constipation and emptying the resident’s bowels became the most important aim of preventive tasks and nursing interventions. Although some preventive measures were taken, FI was mainly treated passively with pads. No interventions were used for most of the residents with diarrhoea or loose stool. The RNs performed repetitive, but not often successful tasks in managing the residents’ bowels. This calls for a new approach for bowel management for older residents in nursing homes. In the context of an increasing ageing population in western countries, investing in the professional development of registered nurses and physicians within the field of incontinence and bowel management may yield significant improvements in dignified and effective care practices. This however requires an awareness of the problem, as well as a commitment to improvement in care practices amongst institutional authorities.

## Abbreviations

ADL: Activities of daily living
FI: Faecal incontinence
ICI: The International Consultation on Incontinence
RN: Registered nurse
